# Motoric Cognitive Risk Syndrome Using 3-Items Recall Test and Its Association With Falls

**DOI:** 10.1093/geroni/igaa057.762

**Published:** 2020-12-16

**Authors:** Hayoung Shim, Miji Kim, Heeeun Jung, Yuri Seo, Seoyoon Jane Lee, Chang Won Won

**Affiliations:** 1 Kyung Hee University, Seoul, Korea, Seoul, Republic of Korea; 2 College of Medicine, Kyung Hee University, Seoul, Republic of Korea; 3 Graduate School, Kyung Hee University, Seoul, Republic of Korea; 4 Elderly Frailty Research Center, Kyung Hee University, Seoul, Republic of Korea; 5 Kyung Hee University Medical Center, Seoul, Korea, Seoul, Republic of Korea

## Abstract

Motoric cognitive risk (MCR) syndrome is defined by the presence of subjective cognitive complaints (SCCs) and slow gait. Its components have synergistic effects to predict various adverse health outcomes. However, some studies reported SCCs might be influenced by cultural differences. Therefore, we devised another criterion to assess the cognitive aspect of MCR to enhance its utility irrespective of the cultural background of the subject by examining the association of MCR and its components with fall-related outcomes. This cross-sectional analysis included 2,641 community-dwelling older adults aged 70-84 years from the Korean Frailty and Aging Cohort Study. These participants did not have dementia and had no difficulties in performing activities of daily living. The newly devised criterion for cognitive aspects of MCR is based on three items recall test on Mini-Mental State Examination. One-hundred-ninety participants (7.2%) met the criteria of MCR using three items recall test. Unlike MCR using SCCs, newly defined MCR showed synergistic effects with fall-related outcomes. Participants with MCR using three items recall test showed a higher risk for falls in the past 1-year (odds ratio [OR] 1.59, 95% confidence interval [CI] 1.08-2.34), recurrent falls (OR 1.86, 95% CI 1.10-3.13), fall with injury (OR 1.60, 95% CI 1.10-2.34), fear of falling (OR 2.77, 95% CI 1.90-4.03), and low activities-specific balance confidence (OR 2.86, 95% CI 1.78-4.60) compared to each component, except for fall with fracture. We found MCR with three items recall test helped predict fall-related outcomes in clinical settings regardless of the cultural context.

